# Labeling images with facial emotion and the potential for pediatric healthcare

**DOI:** 10.1016/j.artmed.2019.06.004

**Published:** 2019-07

**Authors:** Haik Kalantarian, Khaled Jedoui, Peter Washington, Qandeel Tariq, Kaiti Dunlap, Jessey Schwartz, Dennis P. Wall

**Affiliations:** a Department of Pediatrics, Stanford University, USA; b Department of Biomedical Data Science, Stanford University, USA; c Department of Mathematics, Stanford University, USA; d Department of Bioengineering, Stanford University, USA

**Keywords:** Mobile games, Computer vision, Autism, Emotion, Emotion classification

## Abstract

Autism spectrum disorder (ASD) is a neurodevelopmental disorder characterized by repetitive behaviors, narrow interests, and deficits in social interaction and communication ability. An increasing emphasis is being placed on the development of innovative digital and mobile systems for their potential in therapeutic applications outside of clinical environments. Due to recent advances in the field of computer vision, various emotion classifiers have been developed, which have potential to play a significant role in mobile screening and therapy for developmental delays that impair emotion recognition and expression. However, these classifiers are trained on datasets of predominantly neurotypical adults and can sometimes fail to generalize to children with autism. The need to improve existing classifiers and develop new systems that overcome these limitations necessitates novel methods to crowdsource labeled emotion data from children. In this paper, we present a mobile charades-style game, *Guess What?*, from which we derive egocentric video with a high density of varied emotion from a 90-second game session. We then present a framework for semi-automatic labeled frame extraction from these videos using meta information from the game session coupled with classification confidence scores. Results show that 94%, 81%, 92%, and 56% of frames were automatically labeled correctly for categories *disgust*, *neutral*, *surprise*, and *scared* respectively, though performance for *angry* and *happy* did not improve significantly from the baseline.

## Highlights

• Autism spectrum disorder (ASD) affects 750,000 American Children under the age of 10.
• Emotion classifiers integrated into mobile solutions can be used for screening and therapy.
• Emotion classifiers do not generalize well to children due to a lack of labeled training data.
• We propose a method of aggregating emotive video through a mobile game.
• We demonstrate that several algorithms can automatically label frames from video derived from the game.

## 1 Introduction

Autism spectrum disorder (ASD) is a neurodevelopmental disorder affecting an individual's ability to communicate and interact with their peers [1]. While symptoms vary, this condition is generally characterized by stereotyped and repetitive behaviors as well as deficits in social interaction and communication ability such as difficulty recognizing facial expressions, making eye contact, and engaging in social activities with peers [2]. In recent years, the incidence of autism has increased; it is now estimated that one in 40 children in the United States are affected by this condition [3]. While there is no cure, an abundance of evidence has demonstrated the positive impact of early intervention on communication skills and language ability [4].

Common approaches to autism therapy include the Early Start Denver Model (ESDM) and Applied Behavior Analysis (ABA). ESDM therapy supports the development of core social skills through interactions with a licensed behavioral therapist with an emphasis on interpersonal exchange and joint activities [5]. Similarly, ABA therapy is an intervention customized by a trained behavioral analyst to specifically suit the learner's skills and deficits [6]. This program is based on a series of structured activities that emphasize the development of transferable skills to the real world. While both treatments have been shown to be safe and effective, early intervention is essential to maximize the benefits of these programs [4], [7].

Despite significant progress in recent years, imbalances in coverage and barriers to diagnosis and treatment remain. Within the United States, it has been observed that children in rural cities receive diagnosis approximately five months later than those in cities [8]. Moreover, children from families near the poverty line receive diagnosis almost a full year later than those from higher-income families. These delays can defer intervention during times of development considered crucial for maximizing the effectiveness of subsequent behavioral interventions [8]. Alternative solutions that can ameliorate some of these challenges can come from digital and mobile tools, many of which are reliant on computer vision technology that has found increasing application in real-time social support and therapy [9], [10], [11], [12].

Emotion classification is an area of computer vision that emphasizes the development of algorithms that produce an emotion label such as *happy* or *sad* given a photo or video frame containing a face using machine-learning techniques. Our prior work, the Superpower Glass Project, has demonstrated the efficacy of real-time emotion classification to autism therapy via the augmented reality wearable, Google Glass. The Glass unit relays emotion cues in real-time to the child, enabling facial engagement and social reciprocity [13], [14], [15]. Others have also explored the use of wearable systems and affective computing as companion tools for social-emotional learning and the use of the recorded videos for defining a process to collect, segment, label, and use video clips from everyday conversations [9], [16].

**Fig. 1:**
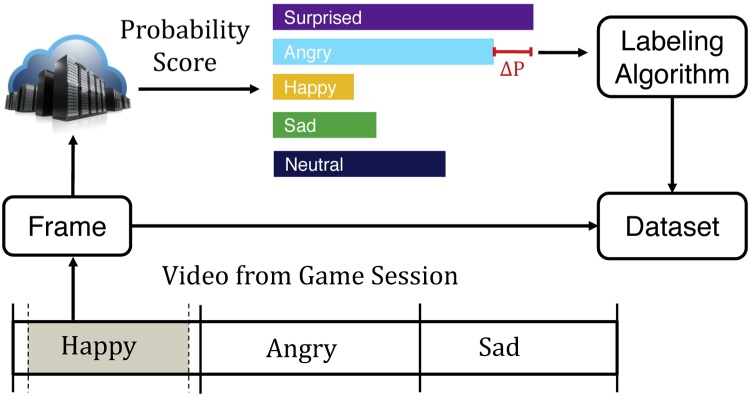
The proposed system is a method to aggregate labeled emotion data from videos derived from a mobile game using classification confidence values and contextual meta-information.
• We present a search algorithm which aims to simultaneously optimize the aggregate number of frames retained as well as the percentage of relevant frames.

A number of emotion classifiers have been developed in recent years by major providers of cloud services including Microsoft Azure Cognitive Services API [17], Amazon Rekognition [18], Google Cloud Vision [19], and others. These algorithms, which typically label an image based on some variation of the seven Ekman emotions [20], are trained on large databases of labeled images such as CIFAR-100 and ImageNet [21]. Datasets specific to facial emotion are also available, such as the Cohn-Kanade Database [22] and Belfast-Induced Natural Emotion Databases [23]. These datasets suffer from a variety of limitations, among which is a lack of generalizability to children: a population significantly underrepresented in these sources. This problem is exacerbated within the domain of autism research, as children with this condition struggle with facial affect and may express themselves in ways that do not closely resemble that of their peers [2], [7]. These variances are unaccounted for in most datasets, rendering some state-of-the-art emotion classifiers unsuitable for vision-based autism research and the development of therapies and assistive solutions derived from these tools. This motivates the development of new approaches for scalable aggregation of emotive frames from children that can be used to design future classifiers and augment existing ones. The primary contributions of this paper are as follows:

• We present a mobile charades-style game, *Guess What?*, designed for a young audience, including those with ASD, from which we can scalably acquire egocentric video with a high density of varied emotion.
• We present a framework for semi-automatic labeled frame extraction from videos derived from *Guess What?* using meta information from the game session coupled with classification confidence scores, shown in Fig. 1.

## 2 Related work

Our primary aim is to develop methods to crowdsource facial-emotion labeled data from children with ASD, with the greater goal of training classifiers suitable for the pediatric population for use as outcome measures, therapies, and screening tools. These systems fall within the scope of *affective computing*: a field that broadly covers the development and application of methods to give computers the ability to recognize and express emotions [24]. An overview of this area was provided in [25], in which Picard described emerging trends in emotion recognition research using electrodermal activity, speech, motion, facial expression, and other sensing paradigms. Picard outlined a vision for future affective computing research that partners psychologists with engineers to interweave emotion detection into everyday life.

Various research efforts have explored whether children with ASD differ in their ability to emote compared to their neurotypical peers. For example, Brewer et al. [26] investigated if individuals with and without ASD can correctly identify emotional facial expressions. The results indicated that regardless of the status of the recognizer, emotions produced by individuals with ASD were more poorly recognized compared to their typically developing peers. By contrast, Faso et al. [27] conducted a study in which 38 observers evaluate the expressions of individuals with and without ASD and showed that ASD expressions were identified with greater accuracy, though they were rated as less natural and more intense compared to those from typically developing individuals. In another study, Capps et al. [28] explored parents’ perceptions of the emotional expressiveness of their children. The findings of this study contradict older studies which suggest an absence of emotional reactions from children with ASD. In fact, the results demonstrated that older children with ASD displayed more facial affect than typically developing children. Other research efforts [29] examine facial muscle movements associated with emotion expression in children with ASD based on videotapes from semi-structured play sessions. This study found that children with autism exhibited reduced muscle movements in certain facial regions compared to typically developing peers.

While several systems have been developed to help children recognize and express facial emotion [14], [30], other studies focused on improving the ability of neurotypical children and adults to interact with individuals with ASD. For example, Tang et al. [31] described an IoT-based play environment designed to allow neurotypical children to better understand the emotions of their peers with autism using a variety of sensors including pressure, temperature, humidity, and a Kinect camera. The authors later conducted a computational study in which they evaluated children's facial expressions during naturalistic tasks in which the children view cartoons while being recorded by a Kinect camera [32]. As before, the aim of this preliminary study was to develop tools to assist typically developing individuals in understanding the emotions of children with autism.

More broadly, Aztiria et al. provided an overview of the field of affect aware ambient intelligence [33]. The authors describe the various forms of affect that can be characterized using wearable and ambient sensors, including voice, body language, posture, and physiological signals such as EEG and EMG. This work provided a broad overview of these techniques as well as several relevant applications such as intelligent tutoring services (ITS)-systems capable of recognizing student affect to assist in the student's learning process. Further work by Karyotis et al. [34] proposed a computational methodology for incorporating emotion into intelligence system design, validated through multiple simulations. The authors proposed a fuzzy emotion representation framework, and demonstrated its utility in big data applications such as social networks, data queries, and sentiment analysis. The work by Maniak et al. [35] proposed a deep neural network model for hierarchical feature extraction to model human reasoning within the context of sound classification.

In recent years, computer vision-based systems have received increasing interest in ASD research. In [36], Marcu et al. proposed a system in which wearable cameras are affixed to children for understanding their needs and preferences while improving their engagement. In [37], Picard et al. provided an overview of methods to automatically detect autonomic nervous-system activation (ASM) in children with ASD to identify and avoid incidents of cognitive overload. Another mobile assistance technology, MOSOC, was presented by Escobedo et al. in [10]. Here, the authors developed a tool that provides visual support of a validated curriculum to help children with ASD practice social skills in real-life situations. These systems are indicative of a general transition from traditional healthcare practices to modern mobile and digital solutions that leverage recent advances in computer vision, augmented reality, robotics, and artificial intelligence. This trend motivates an investigation of methods to augment existing datasets to train new classifiers that generalize to children with ASD.

Several methods of crowdsourced labeled data acquisition have been proposed in recent years. In [38], Barsoum et al. proposed a deep convolutional neural network architecture to evaluate four different labeling techniques. Specifically, the authors explored techniques to combine scores from ten raters into a final label for each image while minimizing errors. Other research efforts [39] have also explored the efficacy of multi-class labels for each image to mitigate the impact of ambiguities on data labeling. In [40], Yu et al. demonstrated that an ensemble of deep learning classifiers can significantly outperform a single classifier for facial emotion recognition. This approach is similar to our own ensemble method, though our technique fuses minimum likelihood with game meta information rather than assigning the label with the maximum probability. This technique, which used variations in probability scores to search for relevant frames and regions within time-series data are inspired partially by prior work on time-series segmentation [41], [42].

## 3 System architecture

**Fig. 2:**
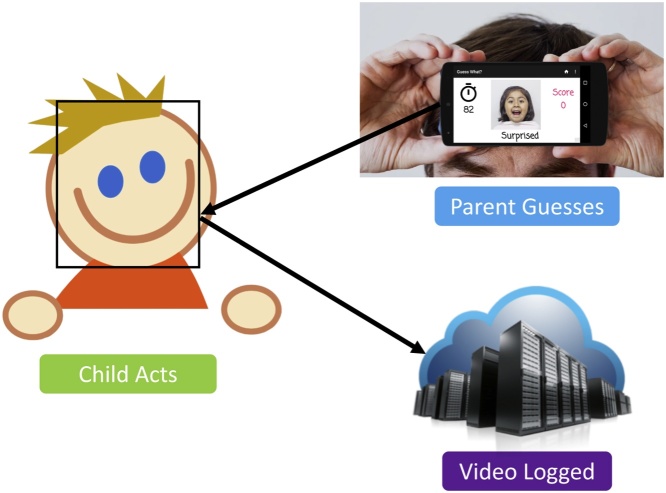
The mechanism for crowdsourcing emotion-labeled frames is a mobile charades game available for Android devices.

*Guess What?* [12] is a mobile Android application modeled after the popular charades game, *Heads Up*. This social gaming activity is shared between the child, who attempts to act out the prompt shown on the screen, and the parent, who holds the phone up to record the child and attempts to guess the word associated with the prompt. This interplay is shown in Fig. 2: the parent positions the phone with the screen facing outward for the entirety of the 90 s game session, as the front camera records the child tasked with representing the prompt using a combination of gestures and facial expressions. The prompt consists of an image with an associated word displayed at the bottom. While several categories of prompt are supported, the two most germane to emotion recognition and expression are *emoji*, which shows exaggerated cartoon representations of emotive faces, and *faces*, which shows real photos of children.

The parent can change the prompt by tilting the phone forward, which awards a point. This occurs when the child acknowledges the correct guess, or in some cases, when the parent makes the determination that the prompt has been represented correctly based on *a priori* knowledge about the image shown. By tilting the phone backward, the prompt is skipped without awarding a point. Immediately thereafter, a new prompt is randomly selected until the 90 s have elapsed. After the game session, parents can review the footage and elect to share the data by uploading the video to an IRB-approved secure Amazon S3 bucket that is fully compliant with Stanford University's High-Risk Application security standards. Meta information is included with the video, which describes the prompts shown, timing data, and the number of points awarded. Using this method of crowdsourced at-home video acquisition, we are developing a database of children with ASD as well as neurotypical children as they express themselves in response to various stimuli.

**Fig. 3:**
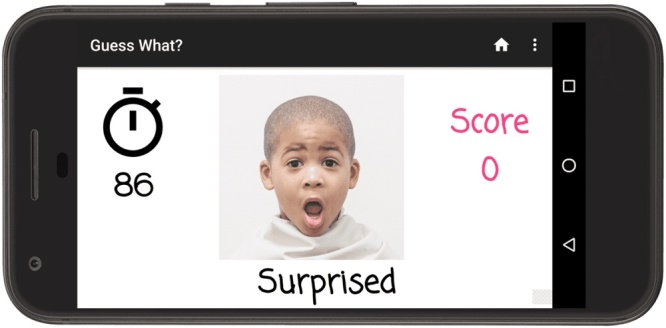
A game session of *Guess What?* in which the child is recorded while acting out the prompt shown on the screen.

An example of the main game screen is shown in Fig. 3: the prompt is shown in the center, with the amount of time remaining displayed on the left and the number of points awarded on the right. This particular prompt is associated with the *faces* category, which is among the most efficacious at deriving emotive facial expressions from children. By contrast, the *animals* category emphasizes vocalizations and *sports* is associated with gestures.

## 4 Algorithms

Videos derived from *Guess What?* can be analyzed frame-by-frame by manual raters to assign emotion labels to each image. However, this approach is tedious and presents an impediment to the scalability of a crowdsource-based system for aggregation of emotive video. In this section, we present several strategies for scalable aggregation of labeled frames from *Guess What?* game sessions using automatic or semi-automatic techniques that leverage both the video and the accompanying meta information from the game session.

### 4.1 Boundary-based segmentation

**Fig. 4:**
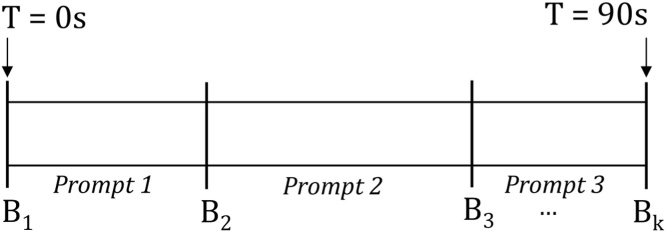
The structure of a single video is characterized by its boundary points, *B*_*i*_ through *B*_*k*_, which identify the times at which various prompts were shown to the child.

The structure of a *Guess What?* video is shown in Fig. 4. Meta information uploaded after each game session delineates the video into regions at which various prompts were shown. For example, frames associated with times at which *Prompt 2* was displayed to the child can be found between timestamps *B*_2_ and *B*_3_. If *Prompt 2* is an emotion-related image, this approach is a reasonable starting point to automatically obtain labeled frames associated with this emotion. More formally, for each emotion we are interested in every frame *f* between a boundary point *b*_*f*_ and the subsequent boundary, *b*_*f*+1_, at which the emotion of the boundary, *e*(*b*_*f*_), matches the emotion we wish to extract, *label*. These conditions are expressed in Eq. (1), where *t* is a function that returns the time associated with a frame or boundary point.

(1) $$\begin{array}{cc} \forall f\;\epsilon \;\mathrm{video}|(t(b_{f})\leq t(f)\leq t(b_{f+1}))\wedge & \\ \;(\mathrm{label}=e(b_{f})) & \end{array}$$

While regions contain a preponderance of emotive frames associated with the prompt shown during this interval, it is unlikely that young children will consistently emote the appropriate emotion during the entirety of the game session. This is particularly true for children with developmental delays who may struggle to recognize, interpret, and convey emotion. Moreover, children may misunderstand their parent's instructions or lose interest in continuing the game session. This motivates additional optimizations to further increase the percentage of retrieved frames that match the emotion of interest. Notwithstanding the possibility of further refinements, this approach in its current form will generally suffice for semi-automatic labeling approaches: scenarios in which the algorithm retrieves a set of likely frames and manual raters filter out incorrect matches.

### 4.2 Sub-bound analysis

While the representation of a video's structure shown in Fig. 4 provides a rudimentary method of identifying high-density regions of various emotions, this model is too simplistic. In practice, there is an interval *α* between the time when the prompt changes and the child's face adjusts accordingly. During this interpretation period, a child will analyze the provided prompt as their face transitions from a typically neutral or happy expression to one associated with the prompt. In theory, complex prompts will require more time for interpretation than the simpler ones: this parameter varies both between subjects and prompts.

**Fig. 5:**
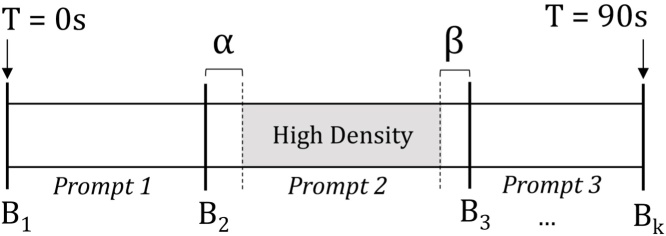
The density of emotion within the video is highest if the leading and trailing frames of the boundary region, *α* and *β*, are cropped.

If the child has correctly represented the prompt, there is a time period *β* before the beginning of the next prompt when the frames are of little use. There are two possible reasons why these frames are best excluded from our analysis. First, the child's face may naturally return to a resting pose in anticipation of the next prompt. Second, the game mechanics of *Guess What?* require the parents to tilt the phone in acknowledgement of a correct guess. In practice, the act of tilting may cause the child's face to briefly leave the frame. The video structure that considers these *α* and *β* parameters is shown in Fig. 5.

Unlike the previous scenario, we are now interested in every frame *f* between a boundary point *b*_*f*_ + *α* and the subsequent boundary, *b*_*f*+1_ − *β*, at which the emotion of the prompt shown in the region, *e*(*b*_*f*_), matches the emotion of the frame we wish to extract, *label*. These conditions are expressed in Eq. (2), where as before, *t* is a function that returns the time associated with a frame or boundary point.

(2) $$\begin{array}{cc} \forall f\;\epsilon \;\mathrm{video}|(t(b_{f})+\alpha \leq t(f)\leq t(b_{f+1})-\beta ) & \\ \wedge \;(\mathrm{label}=e(b_{f})) & \end{array}$$

Algorithm 1 Boundary search algorithm.

**Figure:**
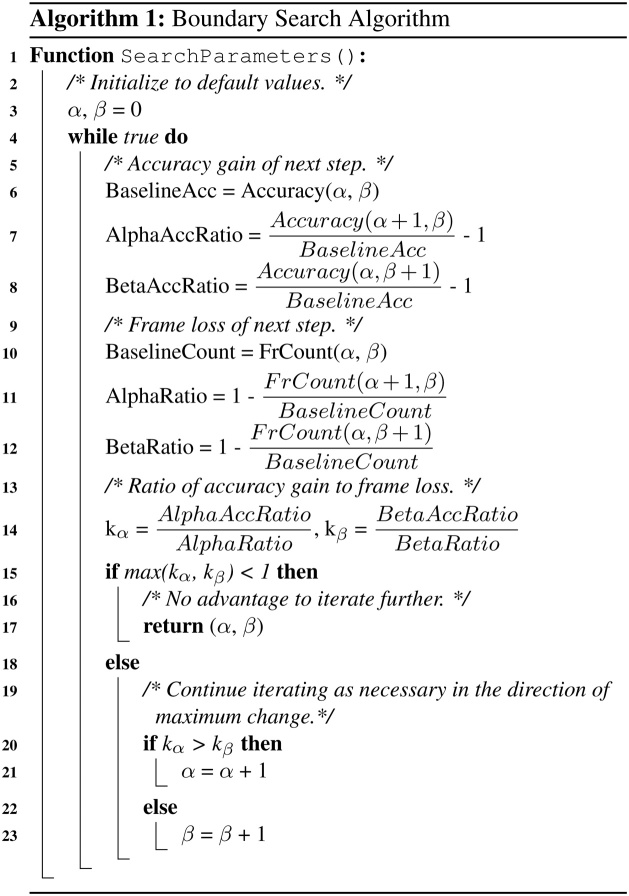

However, increasing the *α* and *β* parameters excessively has the potential to discard potentially relevant frames while offering only marginal improvements to emotion density. We have devised an algorithm to account for these two tradeoffs, which is shown in simplified form in Algorithm 1. The algorithm is initialized with default values, *α* = 0 and *β* = 0. During each step, we evaluate the effects of incrementing *α* and *β* on the increase in percentage of relevant frames and decrease in total number of available frames, the ratio of these two parameters being denoted by *k*. A value of *k* = 1 indicates that the accuracy improved from the baseline by the same margin that the number of frames decreased, which for our application is an acceptable tradeoff. A value of less than 1 suggests marginal improvements to accuracy or perhaps a regression, which is the terminating condition for this algorithm. It should be noted that this algorithm is run on a class-by-class basis to determine optimal *α* and *β* values for each prompt. This decision is motivated by the observation that more complex prompts will require more time to interpret and correctly emote.

### 4.3 Minimum confidence

While the sub-bounds search technique outlined previously can further increase the percentage of frames that match the prompt by filtering out those in the periphery of the region, it remains unlikely that the remaining frames will be associated with the same category as some children may fail to correctly interpret or represent the prompt even within the center of region. This is particularly true for non-trivial prompts that are challenging for children with developmental delays. To further filter out incorrect prompts within the highest-density region with limited manual burden would require an automatic system that can determine if a frame matches the prompt shown to the child. Clearly, no such system exists, due to the lack of labeled data that motivates this work.

**Fig. 6:**
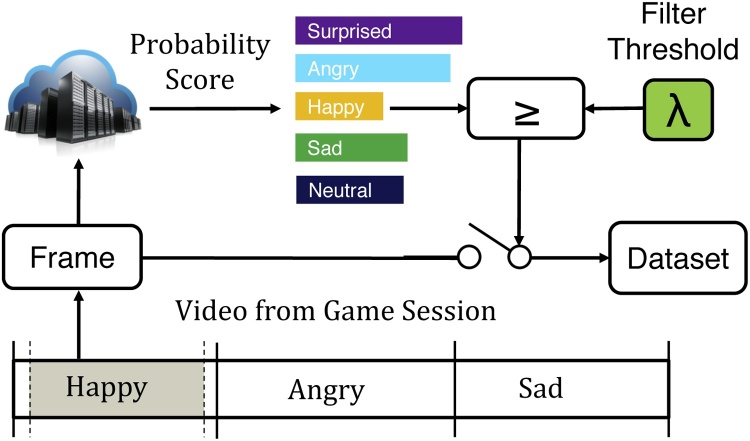
To further improve the percentage of correctly labeled frames, we retain all frames within the region of interest that have a minimum classification confidence of *λ*.

To overcome the limitations of existing emotion classifiers while still leveraging their capabilities, we propose a system in which the classification confidence of the emotion associated with the currently shown prompt acts as a filtering mechanism to eliminate irrelevant frames within the region of interest. While the performance of the classifier is insufficient for us to exclude a frame in which the emotion with the highest classification confidence is discordant with our *a priori* knowledge of the displayed prompt, an extremely low confidence score may still be sufficient grounds for exclusion. This approach is shown in Fig. 6; frames are retained only when located within the highest density region, and when the emotion classifier indicates that the probability of agreement between the emotion in the frame and that of the region exceeds *λ*. Using the same notation as before, Eq. (3) formalizes our approach for retaining frames associated with a specific category, *label*.

(3) $$\begin{array}{cc} \forall f\;\epsilon \;\mathrm{video}|(t(b_{f})+\alpha \leq t(f)\leq t(b_{f+1})-\beta ) & \\ \wedge \;(\mathrm{label}=e(b_{f})) & \\ \wedge \;(\mathrm{Pr}(f=e(b_{f}))\;>\;\lambda ) & \end{array}$$

To obtain *Pr*(*f* = *e*(*b*_*f*_)), the probability that the frame matches the emotion of the prompt shown within this region, we use the Azure Faces API [17] provided by Microsoft Azure Cognitive Services. Given an image transmitted via HTTP request, this API returns an HTTP response containing JSON formatted information about the classification confidences associated with each supported emotion, between 0 and 1. It is important to individually determine *λ* for each class, as classifier sensitivities may be carefully tuned to account for class priors in naturalistic settings that do not generalize to mobile gameplay. This approach, shown in Algorithm 2, is similar to the optimization problem for *α* and *β*; as before, we attempt to optimize the density of relevant frames within the region while avoiding significant decreases in the total number of relevant frames by using the ratio of these two parameters as the terminating condition for the iterative algorithm that returns the final *λ* for each emotion class.

Algorithm 2 Min. confidence algorithm.

**Figure:**
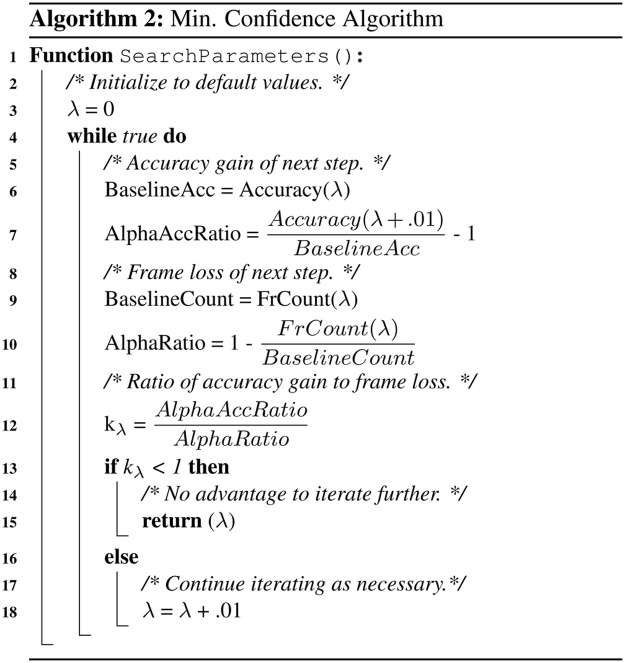

### 4.4 Ensemble classification

A limitation of the previous method is that this technique is too tightly coupled to the nuances of a particular classifier. While indication of a non-zero likelihood of a certain emotion within a frame can be efficacious for making a determination to filter or retain a frame, it is also possible that a classifier reports a 0% likelihood for an emotion that is clearly within the frame. By using classification confidence scores from multiple classifiers, the impact of these anomalies can be mitigated; each classifier's unique nuances can be effectively averaged out to improve the robustness of our filtering algorithm.

**Fig. 7:**
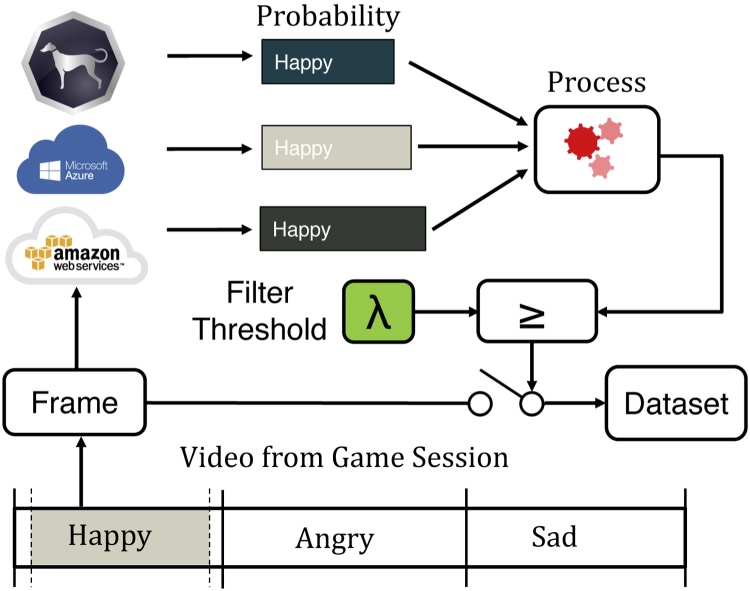
Architecture of the ensemble classification approach.

This ensemble-based approach, which also leverages the sub-bounds search algorithm described previously, is shown in Fig. 7. In addition to AWS, confidence scores are derived from two additional classifiers: Sighthound [43] and Amazon Rekognition [18]. Given three sets of classification confidence scores that are normalized between 0 (minimum confidence) and 1 (maximum confidence), several simple methods can be employed to combine this information into a single value that will be compared to *λ* to make a final filtering decision.

• **Max:** Selecting the maximum classification confidence from all three classifiers for the emotion of interest is a viable choice for classifiers tuned for high precision and low recall.
• **Min:** Selecting the minimum classification confidence from all three classifiers for the emotion of interest is suitable for classifiers tuned for high recall and low precision.
• **Average:** A non-weighted average would be suitable to smooth out the precision/recall biases without requiring careful characterization of their performance.

Regardless of the approach used, the combined confidence score for each of these three techniques would be compared to a class-specific *λ* value.

## 5 Experimental methods

While our long-term objective is to deploy *Guess What?* as a system for crowdsourcing video, an in-lab study provided the data necessary to validate our framework for automatic labeled data extraction. In this section, we describe our methods to obtain the video that formed the basis of our experiments.

### 5.1 Data collection

The dataset used in our experiments was derived from a prior study which included eight children with a prior diagnosis of ASD. The children each played several *Guess What?* games in a single session administered by the same member of our research staff. The average age of participating children with ASD was 8.5 years ±1.85, as shown in Table 3. Due to the non-uniform incidence of autism between genders [1], [44] and small sample size, all participants in this study were boys. During each session, the participant played up to five games with the following decks in no particular order: emoji, faces, animals, sports, and jobs. However, we focus this study on the category most strongly correlated with facial affect, *faces*, which produced a total of 1080 frames.

**Table 3:** List of subjects.

Subject ID	Age	Gender	Diagnosis
1	9	Male	ASD
2	7	Male	ASD
3	6	Male	ASD
4	8	Male	ASD
5	8	Male	ASD
6	12	Male	ASD
7	10	Male	ASD
8	8	Male	ASD

### 5.2 Data processing

Two raters annotated frames to establish a ground truth to evaluate our automatic labeling algorithms: one student (age 23) and one Postdoctoral Researcher (age 29). Both raters were male, and neither had received any relevant clinical training at the time. An important design decision made during this study was to use non-expert raters: those without clinical experience. The motivation for this decision was twofold. First, prior literature has demonstrated that there may be fundamental differences in how children with autism express emotions, which could affect the ability of individuals to recognize and perceive facial emotion from children with developmental delay [26], [29]. Building a dataset of emotion-labeled frames understandable to clinicians but not by the general population could be detrimental to our long-term objective of building AI-enabled systems to help children develop their ability to communicate with their *peers*-rather than those with clinical training. Additional factors that motivated this decision were the conclusion drawn from our prior work [45], which demonstrated that raters without clinical expertise are capable of annotating videos from children with developmental delay with high sensitivity and specificity. These findings are corroborated by the high inter-rater reliability scores between the two raters used in this study, as shown in Fig. 10.

The raters manually assigned emotion labels to each frame in the selected videos based on the seven Ekman universal emotions [20] with the addition of a *neutral* class. In cases when no face could be located within the frame, or the frame was too blurry to discern, reviewers did not assign a label. To simplify annotation and establish a format consistent with commercial emotion classification APIs, the *anger* and *contempt* emotions were merged into a single category. Furthermore, the *confusion* emotion was ignored as not every emotion classifier supported it and no related prompts were shown during these game sessions. A total of 1350 frames were manually labeled by the two raters. Frames were discarded in cases when the raters disagreed or did not assign a label. This produced a total of 1080 frames from the original 1350, distributed between emotions as shown in Table 2.

**Table 2:** Total frames per category.

Category	Frames
Total	1080
Neutral	506
Non-neutral	574
Happy	167
Sad	104
Surprised	127
Scared	28
Disgusted	118
Angry	30

The number of frames both manual raters assigned to the same category, for the dataset used in our experiments.

## 6 Results

In this section, we describe the accuracy of our proposed automatic labeling techniques as well as the inter-rater reliability for the manual annotation that served as the ground truth of our experiments.

### 6.1 Inter-rater reliability

From a total of 1350 frames, 1185 were flagged as valid: frames which both raters agreed were of sufficiently high quality to assign an emotion label. From these 1185 valid frames, the raters assigned the same emotion to 1080 (91%). The Cohen's Kappa statistic for inter-rater reliability, a metric which accounts for agreements due to chance, was 0.10. This indicates a high level of reliability between the two manual raters.

Fig. 10 shows the distribution of frames between the manual raters, for all valid frames. Most misclassified frames were between the happy-neutral and sad-neutral categories. The abbreviations used in this figure are defined in Table 1.

**Table 1:** Abbreviations

	Emotion
HP	Happy
SD	Sad
AG	Angry
DG	Disgusted
NT	Neutral
SC	Scared
SP	Surprised

Abbreviations for emotions used throughout this paper.

### 6.2 Distribution of frames

Table 2 shows the total number of frames in each category from all three videos, omitting those frames in which the manual raters disagreed on the label. Frames that are designated as non-neutral refer to those valid frames which have a label other than the *neutral* class. From the 1080 total frames, 46.8% were neutral compared to 53.1% non-neutral frames. The most represented emotion was *happy*, with 167 frames, followed by *surprised* and *disgusted* with 127 and 118 frames respectively. The two least represented emotions were *scared*, with 28 frames, and *angry*, with 30.

### 6.3 Baseline: boundary analysis

**Fig. 8:**
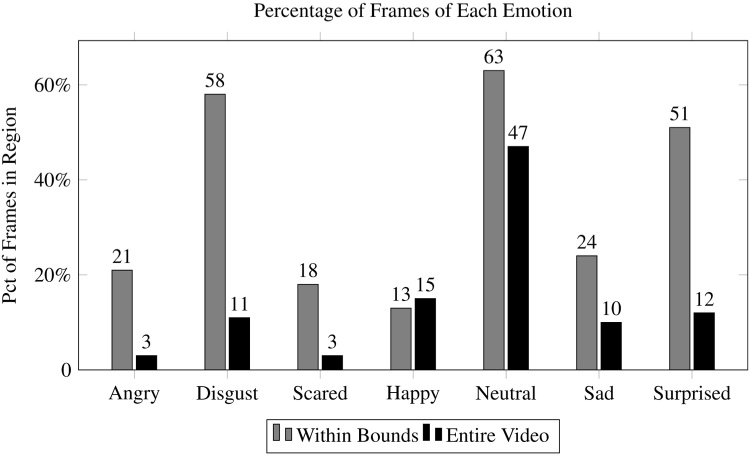
A much higher percentage of frames for a given emotion can be found during the times in which the associated prompt was shown on the screen, compared to their prevalence throughout the entire video. This is particularly true for prompts that are otherwise sparse, such as *angry* and *scared*.

**Fig. 9:**
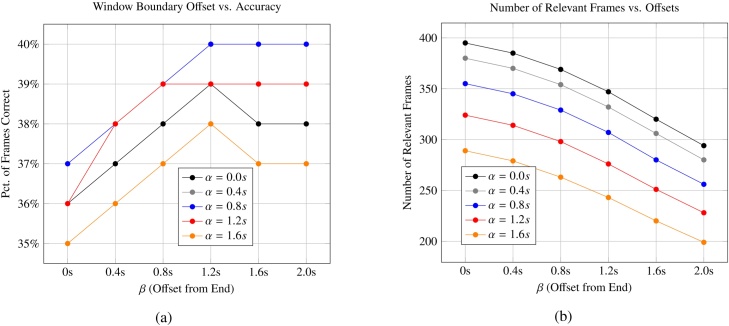
(a) Parameter *α* refers to the number of frames (at 5 frames per second) skipped at the beginning of the window, while *β* refers to the number of frames omitted before the end of the window. (b) As parameters *α* and *β* are tuned to increase the percentage of correct frames within the boundary, the total number of frames may decrease.

Fig. 8 provides a visualization of the percentage of frames within the boundary region that matched the emotive prompt shown during these times, based on three 90-second video sessions from three children subsampled to five frames per second. While the majority of frames within the *disgust* and *neutral* region matched the prompt, performance was poor for *happy* and *scared*. As shown in Fig. 9, regions contained a much higher percentage of relevant emotions compared to the prevalence of these emotions throughout the entire video. Moreover, the videos derived from *Guess What?* contained a reasonable diversity of emotive frames from various categories as shown in Table 2. Naturally, some emotions were more sparsely represented than others; *scared* and *angry* were associated with 28 and 30 frames, respectively. However, these disparities can be rectified by modifying the composition of prompts to emphasize these less common emotions.

**Fig. 10:**
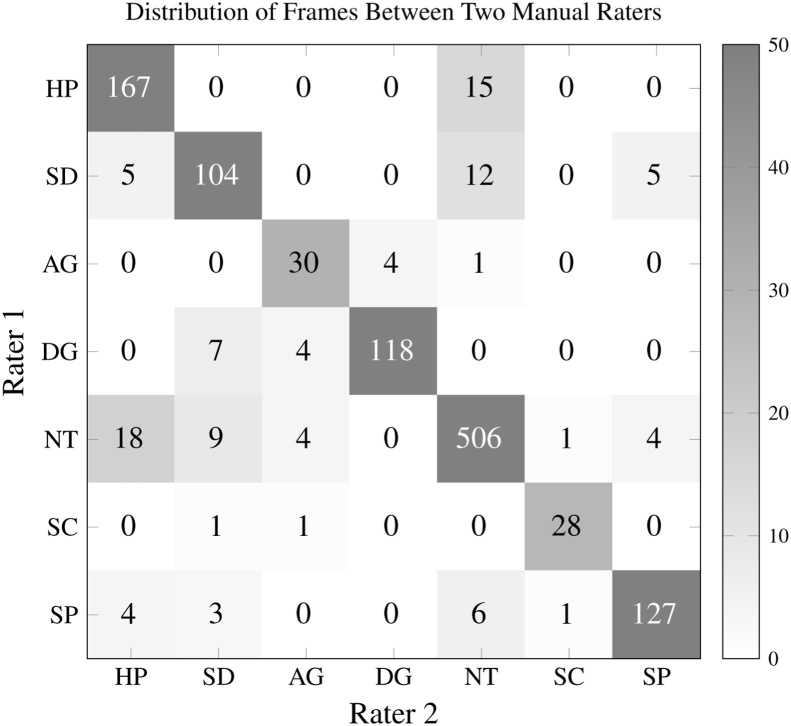
The confusion matrix of the two raters assignments of frames into emotion categories.

### 6.4 Sub-bound analysis

Results suggest that the central region of the boundary generally has a higher density of relevant frames. Fig. 9A shows the percentage of frames which match the emotion associated with the region as a function of *α* and *β*, when optimizing globally rather than on a per-emotion basis. Baseline accuracy was approximately 35%, but increased to 40% with *α* and *β* values of 0.8 s and 1.2 s, respectively.

Fig. 9B shows the raw number of relevant frames retained within a region that matched the boundary as a function of these two parameters. It is important to carefully consider the possibility of loss of frames when tuning these parameters. For example, choosing a *β* value of 2.0 s and an *α* value of 1.6 s reduces the number of relevant frames by over 50%, with only marginal improvements to accuracy.

After optimizing on a per-class basis using Algorithm 1, the value of these parameters is shown in Table 4, and varies widely between prompts. For instance, the *happy* prompt did not require any trimming. This is likely because many children were smiling throughout the game session, irrespective of the prompt shown. The large *α* time associated with the neutral class could be caused by the uncertainty a non-emotive class introduced as most other prompts had a clear and perhaps exaggerated emotion associated with them. The large trailing times for *disgusted* and *surprised* might be explained by the relative discomfort of maintaining these exaggerated facial expressions for extended periods, though a much larger dataset is necessary to draw definitive conclusions.

**Table 4:** Optimal parameters per emotion.

Category	*α*	*β*	*λ*
Neutral	2.2 s	0	0.02
Happy	0	0	0.00
Sad	0.4 s	0.4 s	0.00
Surprised	0.4 s	1.6 s	0.10
Scared	1.0 s	1.0 s	0.00
Disgusted	0.6 s	1.8 s	0.01
Angry	0.4 s	0.6 s	0.00

The number of frames skipped at the beginning and end of the window, *α* and *β*, varied per prompt, as did the minimum classification confidence used to filter frames, *λ*.

**Fig. 11:**
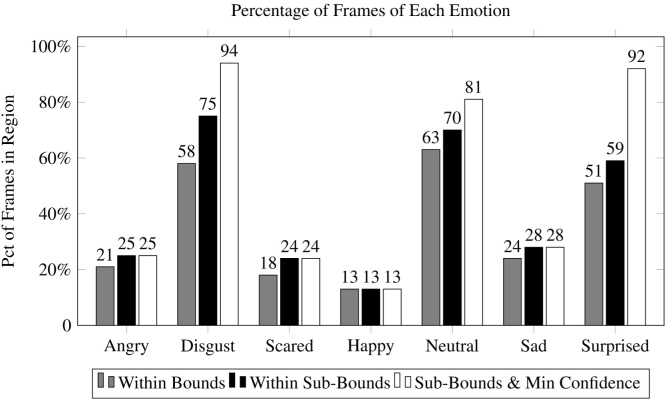
Adjusting the *α* and *β* parameters did not improve the percentage of correctly classified frames for every prompt, but improved accuracy for *scared, neutral* and *surprised*.

**Fig. 12:**
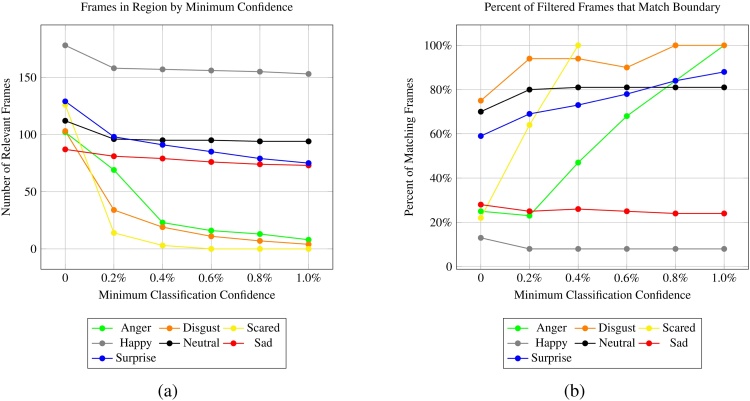
(a) Retaining only frames which the classifier reports to match the emotion associated with the boundary can dramatically reduce the number of remaining frames for various classes. (b) Retaining only frames which the classifier reports to match the class associated with the boundary region can increase the percentage of relevant frames for some emotions.

Fig. 11 shows the percentage of matching frames using the sub-bound approach on a per-class basis, with results from this technique denoted by black bars. For several categories, *disgust*, *neutral*, and *surprise*, the percentage of matching frames increased significantly. The improvement was most pronounced for *disgust*, which increased from 58% to 75%. However, the percentage of relevant frames remained constant for *happy* and improved only marginally for *angry* and *sad* (Fig. 12).

### 6.5 Sub-bound + minimum confidence

The optimal minimum confidence score, *λ* is shown in Table 4 based on results obtained using the Microsoft Azure Cognitive Services API [17] using the search approach shown in Algorithm 2. Recall that *λ* represents the minimum required classification confidence of the the emotion associated with the region in which a frame is found for it to be retained by the filtering algorithm.

The requisite *λ* was very small for every class, ranging from 0.00 (no filtering) for *happy* to 0.10 (10%) for surprise. The improvement derived from this method is likely because the classification confidence reported by the classifier may be too conservative when contextual knowledge indicates that the frame was derived in a region that matches the class associated with the prompt shown. Results for this approach are denoted by the white bars in Fig. 11. The classes that improved from the baseline method to the sub-bound approach increased further using the minimum confidence method: *disgust* increased from 75% to 94%, *neutral* increased from 70% to 81%, and *surprise* increased from 59% to 92%. However, no substantial improvements were found for the other categories.

### 6.6 Sub-bound and ensemble

**Fig. 13:**
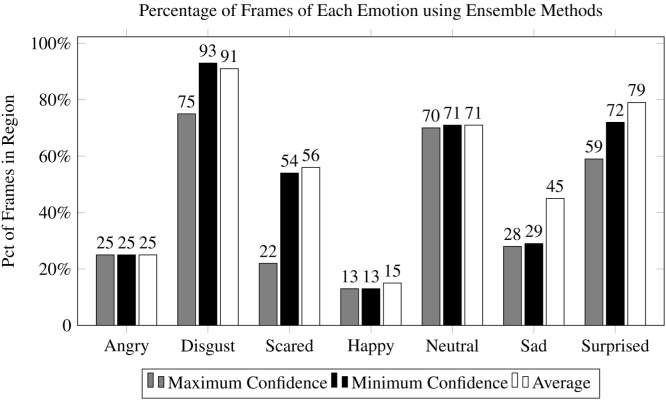
A comparison of three different methods of combining multiple classification confidence scores to make a filtering decision demonstrates that averaging the scores was generally the best technique.

Fig. 13 shows the percentage of frames correctly identified within a region when filtering using an ensemble-based technique that combines classification confidence scores from multiple classifiers using three different methods: minimum, maximum, and average, and comparing the result to a predefined threshold, *λ*. It should be noted that in some cases, the best ensemble-based technique was still outperformed by the minimum-confidence technique using a single-classifier.

• **Max:** Selecting the maximum classification confidence from all three classifiers did not improve performance from the baseline for any emotion. This is likely because the evaluated emotion classifiers provided generally very high confidence scores, even for frames that did not match the desired emotion. The results shown in this figure are associated with a *λ* = 0: no filtering.
• **Min:** When filtering based on the minimum classification confidence score between all three classifiers, the percentage of matching frames within a region increased considerably for *disgust*, *scared*, and *surprised*.
• **Average:** Averaging the confidence score from all three classifiers provided the best overall accuracy, though improvement in the *happy* category was marginal and nonexistent in the case of *angry*.

### 6.7 Discussion

**Fig. 14:**
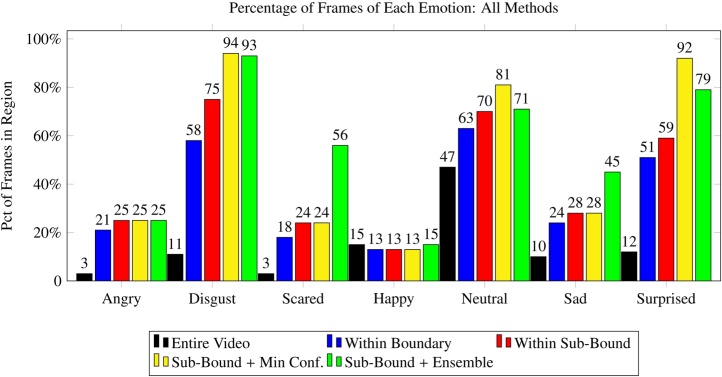
A comparison of the methods described in this work shows that a hybrid minimum-confidence ensemble technique that uses the optimal sub-bound for a region is able to make a correct filtering decision for the majority of frames for four out of the seven evaluated emotions.

Fig. 14 provides a direct comparison of the five techniques used to obtain labeled emotion data in this work, which we briefly summarize here.

• **Entire video:** A baseline method that evaluates the percentage of frames in a video that match a particular class of emotion.
• **Within boundary:** Aggregating frames from regions within the video where the prompt related to the emotion of interest are shown.
• **Within sub-bound:** Searching within the boundary but filtering out leading and trailing frames and limiting the search to the center of the region.
• **Sub-bound + minimum confidence** Searching within the center of the region, and further filtering frames in which the classification confidence of the emotion of interest did not exceed a predefined threshold.
• **Sub-bound + ensemble** Like before, but using multiple classifiers, combining their classification confidences, and comparing the result to a predefined threshold to make a filtering decision.

Results indicate that a high percentage of frames associated with *disgust, scared, neutral,* and *surprised* can be derived using these techniques (94%, 56%, 81%, and 92% respectively), with the ensemble method producing the strongest results overall. This suggests that the provided framework is sufficient for automatic aggregation of labeled frames from some emotions, and semi-automatic labeling of others. However, results for the *angry* and *happy* categories remained poor across all techniques. This shortcoming could be caused in part by few subjects or limited manual raters. Given the ambiguity of exactly when a face transitions from *neutral* to *happy*, the manual raters could have made labeling decisions that were incongruous with the classifier's definition of a happy face. Regardless, additional experimentation and novel techniques are necessary to bridge this gap and provide methods to derive emotive frames from all categories in structured video.

## 7 Limitations and future work

In this work, we propose a method of crowdsourcing emotion-labeled frames from children with Autism Spectrum Disorder using a mobile application and various automatic labeling algorithms. Future work will validate this approach on a larger, more varied dataset. Moreover, we will include a ground truth of manually annotated frames derived from a greater number of raters with clinical experience to determine if there are appreciable accuracy improvements compared to labels from the two raters used in this study. Subsequently, a deep neural network model will be trained using a transfer-learning approach to validate our hypothesis that the limitations of existing systems arise from a lack of relevant training data.

The ecological validity of novel interventions for ASD is an important concern. A conference organized by a multidisciplinary panel of researchers of developmental disabilities developed a list of best practices for screening and early identification of autism in October of 2010 [46]. A significant conclusion drawn from this conference was that intervention research should integrate culturally and socially diverse populations to evaluate factors that influence both the participation and outcomes of therapeutic approaches. Therefore, it is crucial for data collection efforts of follow-up studies to consider cultural contexts outside the United States and to represent a more diverse cohort of children.

## 8 Conclusion

We present a system for deriving emotive video from children with ASD through a charades-style game, and several algorithms that can be used to extract semi-labeled frames from these videos using classification confidence scores and game meta information. We demonstrate three techniques: Sub-Bound Analysis, Minimum Confidence, and Ensemble Classification, that we compare to a baseline method on the basis of their efficacy in correctly labeling frames from videos derived from *Guess What?* game sessions. Results show that 94%, 81%, 92%, and 56% of frames were automatically labeled correctly for categories *disgust*, *neutral*, *surprise*, and *scared* respectively, though performance for *angry* and *happy* did not improve significantly from the baseline. Once additional video data are available, these methods will be employed to generate a large labeled dataset that will be used to train convolutional neural network classifiers for emotion recognition that are robust across differences in age and developmental delay.
